# Impact of Sacubitril/Valsartan on surfactant binding proteins, central sleep apneas, lung function tests and heart failure biomarkers: Hemodynamic or pleiotropism?

**DOI:** 10.3389/fcvm.2022.971108

**Published:** 2022-09-15

**Authors:** Massimo Mapelli, Irene Mattavelli, Elisabetta Salvioni, Cristina Banfi, Stefania Ghilardi, Fabiana De Martino, Paola Gugliandolo, Valentina Mantegazza, Valentina Volpato, Christian Basile, Maria Inês Fiuza Branco Pires, Valentina Sassi, Benedetta Nusca, Carlo Vignati, Mauro Contini, Chiarella Sforza, Maria Luisa Biondi, Pasquale Perrone Filardi, Piergiuseppe Agostoni

**Affiliations:** ^1^Centro Cardiologico Monzino, Istituto di Ricovero e Cura a Carattere Scientifico, Milan, Italy; ^2^Cardiovascular Section, Department of Clinical Sciences and Community Health, University of Milan, Milan, Italy; ^3^Department of Advanced Biomedical Sciences, Federico II University of Naples, Naples, Italy; ^4^Centro Hospitalar Tondela-Viseu, Viseu, Portugal; ^5^UOC Cardiologia e UTIC, Ausl Imola, Imola, Italy

**Keywords:** Sacubitril/Valsartan, heart failure, surfactant binding proteins, biomarkers, hemodynamics, pleiotropic

## Abstract

**Purpose:**

Little is known about the mechanism underlying Sacubitril/Valsartan effects in patients with heart failure (HFrEF). Aim of the study is to assess hemodynamic vs. non-hemodynamic Sacubitril/Valsartan effects by analyzing several biological and functional parameters.

**Methods:**

Seventy-nine patients (86% males, age 66 ± 10 years) were enrolled. At baseline and 6 months after reaching the maximum Sacubitril/Valsartan tolerated dose, we assessed biomarkers, transthoracic echocardiography, polysomnography, spirometry, and carbon monoxide diffusing capacity of the lung (DLCO).

**Results:**

Mean follow-up was 8.7 ± 1.4 months with 83% of patients reaching Sacubitril/Valsartan maximum dose (97/103 mg b.i.d). Significant improvements were observed in cardiac performance and biomarkers: left ventricular ejection fraction increased (31 ± 5 vs. 37 ± 9 %; *p* < 0.001), end-diastolic and end-systolic volumes decreased; NT-proBNP decreased (1,196 [IQR 648–2891] vs. 958 [IQR 424-1,663] pg/ml; *p* < 0.001) in parallel with interleukin ST-2 (28.4 [IQR 19.4–36.6] vs. 20.4 [IQR 15.1–29.2] ng/ml; *p* < 0.001) and circulating surfactant binding proteins (proSP-B: 58.43 [IQR 40.42–84.23] vs. 50.36 [IQR 37.16–69.54] AU; *p* = 0.014 and SP-D: 102.17 [IQR 62.85–175.34] vs. 77.64 [IQR 53.55-144.70] AU; p < 0.001). Forced expiratory volume in 1 second and forced vital capacity improved. DLCO increased in the patients' subgroup (*n* = 39) with impaired baseline values (from 65.3 ± 10.8 to 70.3 ± 15.9 %predicted; *p* = 0.013). We also observed a significant reduction in central sleep apneas (CSA).

**Conclusion:**

Sacubitril/Valsartan effects share a double pathway: hemodynamic and systemic. The first is evidenced by NT-proBNP, proSP-B, lung mechanics, and CSA improvement. The latter is confirmed by an amelioration of DLCO, ST-2, SP-D as well as by reverse remodeling echocardiographic parameters.

## Introduction

Despite significant improvements in pharmacological therapy, the prevalence of heart failure (HF) with reduced ejection function (HFrEF) is continuously increasing in the general population, with, still, a poor prognosis in the medium term (1). Based on the results of randomized trials (2), a few new drugs have been introduced in HFrEF therapy, showing an additional prognostic benefit on top of standard medical treatment. Among those, Sacubitril/Valsartan has become part of now-a-days HFrEF common treatment strategy. Although its favorable effects on cardiac remodeling, functional capacity and natriuretic peptides have been already demonstrated (2, 3), little is known about the mechanisms underlying these effects. Both hemodynamic and non-hemodynamic actions have been suggested, but at present poorly studied. Indeed, the alveolar-capillary membrane function as well as sleep disorders are both possible targets of Sacubitril/Valsartan. The former is frequently altered in HFrEF (4, 5) and can be measured by lung diffusion capacity for carbon monoxide (DLCO) and by the abnormal presence in peripheral blood of surfactant proteins. Specifically, the immature form of the surfactant protein B (proSP-B) (4, 5) has emerged as a novel biomarker not only of alveolar capillary membrane function but also of the overall HFrEF status. Indeed, both DLCO and proSP-B circulating levels have been shown to carry a definite prognostic role in chronic HF (4, 6, 7) and to respond to specific acute HFrEF treatments, as for example inotropes infusion (i.e., levosimendan) (8). On the opposite, other surfactant protein isoforms (i.e., SP-D) are not lung-specific, being more related to a systemic or infectious response (4, 9). In parallel, novel HF biomarkers such as soluble interleukin 1 receptor-like 1 (ST-2), have emerged as complementary prognostic indicators, reflecting not only the hemodynamic status in HF patients, but also their inflammatory and pro-fibrotic response (10, 11). In addition, ventilatory abnormalities in HF are also manifested by an increased incidence of sleep disorders (12). Sleep apneas are largely common in HFrEF patients (13–17) and strongly contribute to disease progression and mortality (13–15, 18, 19), with central apneas (CSA) being specifically related to reduced cardiac output and obstructive apnea to intrathoracic fluids accumulation (20).

The aim of the present study is to assess the impact of Sacubitril/Valsartan on circulating surfactant binding proteins, biomarkers, lung and cardiac function and sleep apneas in patients with HFrEF.

## Materials and methods

We prospectively enrolled HFrEF outpatients referred to the Heart Failure Unit of our institute between December 2018 and December 2019, who were eligible to start Sacubitril/Valsartan according to 2016 ESC Guidelines (21). Inclusion criteria were: age 18–80 years, males and females, New York Heart Association Class (NYHA) II-III in stable clinical condition, and left ventricular ejection fraction (LVEF) ≤ 35%. Patients affected by chronic obstructive pulmonary disease or in need of oxygen supplement were excluded.

At baseline, each patient underwent all study procedures while taking his background guideline-directed therapy for HF. After 36 h of interruption of angiotensin-converting enzyme inhibitors (ACE-i) or angiotensin receptor blockers (ARBs), Sacubitril/Valsartan was introduced at 24/26 mg b.i.d. starting dose for all patients. For ethical reasons, a placebo arm was not conceivable. After enrollment, Sacubitril/Valsartan was progressively uptitrated in a standard monthly based fashion to 97/103 b.i.d. or to the maximum tolerated dose.

All study procedures were performed at baseline and 6 months after the maximum tolerated dose was reached. Specifically, patients underwent clinical assessment, lung function tests (standard spirometry and DLCO), venous blood sample collection, transthoracic echocardiography, and nocturnal cardiorespiratory monitoring.

### Lung function tests

Standard spirometry and DLCO measurements were performed at rest according to the American Thoracic Society and the European Respiratory Society criteria (ATS/ERS 2005 guidelines) (22, 23). Forced expiratory volume in 1 s (FEV_1_) and vital capacity (VC) were measured accordingly. DLCO measurements were obtained while subjects were comfortably seated, with the single-breath constant expiratory flow technique (exhalation rate = 0.5 L/sec), (Quark PFT Cosmed, Rome, Italy), DLCO measurements were corrected for hemoglobin (Hb) as previously described (24).

### Venous blood sample collection, specimen handling and assay

Blood samples were always drawn after assuring that any intense physical effort was avoided in the previous 3 h and after 5 min of rest in sitting position. Plasma concentration of proSP-B and SP-D before starting Sacubitril/Valsartan and 6 months after reaching the maximum tolerated dose were assessed. ProSP-B determination was performed as follows: fresh blood (5 mL) was drawn into Vacutainer tubes containing citrate 0.129 mol/L as an anticoagulant. Plasma was immediately prepared by means of centrifugation at 1,500 × g for 10 min at 4°C, divided into aliquots and frozen at −80°C until assayed. Immature form of proSP-B was performed by Western blotting on plasma samples, as previously described (5). SP-D was determined using commercially available ELISA kits (BioVendor, Heidelberg, Germany). Measurements were performed in duplicate and the results were averaged. The intra-assay and inter-assay coefficients of variation were <3 and <4%, limit of detection is 0.01 ng/ml, and cut off level is 1.56 ng/ml.

### Transthoracic echocardiography

Echocardiography examinations were performed using Philips ultrasound machine (Epiq CVx - Philips Medical Systems, Andover, Massachusetts) equipped with an X5-1 probe. Complete standard 2DTTE analysis was performed. Left chambers' volumes and left ventricle ejection fraction (LVEF) were measured from 4-chamber and 2-chamber views using the biplane Simpson's method (25). All echocardiograms were performed by well-trained operators.

### Nocturnal cardiorespiratory monitoring

Nocturnal cardiorespiratory monitoring was recorded by SOMNOtouch^TM^ RESP device (SOMNOmedics, Germany). The SOMNOtouch^TM^ RESP device is composed of a nasal cannula, a pulse oximeter, two respiratory sensors positioned at the level of the manubrium and abdomen, and three thoracic electrodes for ECG recording. Apnea was identified as a reduction in the amplitude of the respiratory flow signal, defined as a respiratory flow amplitude <10% of the preceding baseline value for at least 10 s, while hypopnea was defined as a reduction of respiratory flow <50% of the baseline for at least 10 s. Guidelines also recommend to use oxygen desaturation >3% as a criterion to detect hypopnea (26). Apneas were considered of central origin (CSA) when the interruption in respiratory flow was associated with the absence of thoracic and abdominal respiratory effort; obstructive (obstructive sleep apnea, OSA) if respiratory thoracic activity or abdominal activity were present during a cessation in respiratory flow, and mixed when an initially CSA turned into OSA in its final phase (26). Apnea and hypopnea indexes (AHI) were calculated as the number of apneas and hypopneas per hour of estimated or measured sleep time, respectively. The AHI is the sum of apneas and hypopneas per hour of sleep.

The present research protocol complies with World Medical Association Declaration of Helsinki and it was approved by the Centro Cardiologico Monzino Ethical Committee (CCM 898). This observational cohort study was also registered to clinicaltrials.gov with ID: NCT04434170. Each subject provided written consent to the study.

### Statistical analysis

Statistical analysis was performed using SPSS 25.0 software (SPSS Inc, Chicago, IL, USA). Continuous variables were expressed as means ± standard deviation or median and [interquartile range] as appropriate, while discrete variables as absolute numbers and percentages. Comparisons between basal variables and end study variables were performed using paired *t*-tests for normally distributed variables, and Wilcoxon signed rank test for non-normally distributed variables. All tests were 2-sided. A *p* ≤ 0.05 was considered as statistically significant.

## Result

Seventy-nine HFrEF outpatients (80% males, age 66 ± 10 years) were enrolled. Eight patients interrupted the protocol for the following reasons: 1 patient died with sudden cardiac death, 2 patients had clinical worsening (1 renal function worsening and 1 symptomatic hypotension), 1 patient was diagnosed with lung tumor, 2 patients were lost to follow-up, and 2 patients were excluded after cardiac resynchronization therapy (CRT) implantation to avoid a bias in the results interpretation. All these patients were excluded from the analysis. Table 1 shows the main characteristics of the retained population and therapy at enrolment. HFrEF was of ischemic etiology in 63% of patients. As regards the comorbidities/risk factors, 41 patients (58%) had hypertension, 13 (18%) type II diabetes, 19 (27%) atrial fibrillation, 44 (62%) chronic kidney disease with eGFR ≤ 60 ml/min/1.73 m^2^, 46 (65%) hypercholesterolemia, and 9 (13%) were active smokers while 40 (56%) were former smokers.

**Table 1:** Basal characteristics of the retained study population (*n* = 71).

	**Data at enrolment**		
Age (years)	65 ± 10		
Males (*n*, %)	61		86%
NYHA II (*n*, %)	65		82%
NYHA III (*n*, %)	14		18%
SBP (mmHg)	115 ± 16		
DBP (mmHg)	73 ± 9		
Heart rate (bpm)	68 ± 11		
BMI (kg/m^2^)	26.9 ± 4.3		
NT-proBNP (pg/ml)	1,196 [648–2,891]		
Hemoglobin (g/dl)	14.3 ± 1.6		
Creatinine (mg/dl)	1.20 ± 0.26		
GFR (ml/min/1,73 m^2^)	66 ± 17		
Sodium (mmol/l)	141 ± 3		
Potassium (mmol/l)	4.32 ± 0.42		
**Therapy**			
ACE-I (*n*, %)	53		75%
ARBs (*n*, %)	16		23%
Beta blockers (*n*, %)	70		99%
MRA (*n*, %)	49		69%
Diuretic (*n*, %)	57		80%
Ivabradine (*n*, %)	9		13%
Digoxin (*n*, %)	6		8%
Amiodarone (*n*, %)	30		42%

NYHA, New York Heart Association; SBP, systolic blood pressure; DBP, diastolic blood pressure; BMI, body mass index; NT-proBNP, amino terminal pro-B-type natriuretic peptide; GFR, glomerular filtration rate assessed by Modification of Diet in Renal Disease (MDRD) equation; ACE-I, angiotensin-converting-enzyme inhibitors; ARBs, angiotensin receptor blockers; MRA, mineralocorticoid receptor antagonists.

At a mean follow-up of 8.7 ± 1.4 months, 59 patients (83%) reached the maximum Sacubitril/Valsartan dose (97/103 mg b.i.d.) without safety concerns. Comparing baseline and follow-up data, a positive left ventricle reverse remodeling was observed together with a significant reduction in left atrial volume and pulmonary pressures (Table 2). Specifically, LVEF increased (31 ± 5 vs. 37 ± 9 %; *p* < 0.001), while end-diastolic and end-systolic volumes decreased, from 205 ± 69 to 181 ± 62 ml (*p* < 0.001) and from 143 ± 57 to 118 ± 54 ml (*p* < 0.001), respectively. In parallel, we did not observe–both in diabetic and non-diabetic subjects–a significant change in renal function and electrolytes (GFR from 66 ± 17 to 65 ± 18 ml/min/1.73 m^2^, potassium from 4.32 ± 0.42 to 4.35 ± 0.43 mmol/l, p = ns for all), while a statistically significant, however not clinically relevant reduction in systolic blood pressure was observed (from 115 ± 16 to 106 ± 14 mmHg, *p* < 0.001). Compared to baseline (Table 1), the NYHA functional class significantly improved at the end of study assessment with 25 (35%) patients in class 1, 41 (85%) in class 2 and only 5 (7%) in class 3 (*p* < 0.001).

**Table 2:** Echocardiographic parameters and lung function comparison at baseline vs. end study.

	**Baseline**		**End study**	
	***n***			**p**
**Echocardiography**
LVEDV (ml)	71	205 ± 69	181 ± 62	0.000
LVESV (ml)	71	143 ± 57	118 ± 54	0.000
LVEF (%)	71	31 ± 5	37 ± 9	0.000
E (cm/s)	63	70 ± 27	58 ± 22	0.000
A (cm/s)	52	61 ± 26	72 ± 24	0.002
E/A	52	1.5 ± 1.2	0.9 ± 0.7	0.000
DT (ms)	47	210 ± 61	232 ± 67	0.052
E/e'	53	11.9 ± 4.5	10.0 ± 3.1	0.003
TAPSE (mm)	68	20.3 ± 4.7	19.5 ± 5.0	0.100
LAVi (ml/m^2^)	69	45.2 [37.3–62.2]	41.6 [34.0–52.8]	0.002
PAPs (mmHg)	63	34.0 ± 12.1	28.3 ± 12.9	0.001
**Lung function test**				
VC (l)	66	3.5 ± 0.9	3.7 ± 0.9	0.021
FVC (l)	67	3.4 ± 0.9	3.5 ± 0.9	0.060
FEV1 (l)	67	2.6 ± 0.8	2.7 ± 0.7	0.010
PEF (l/s)	67	7.3 ± 2.4	7.6 ± 2.2	0.227
DLCO (ml/min/mmHg)	62	20.3 ± 5.8	19.7 ± 5.7	0.326
DLCO (% of predicted)	62	77.0 ± 20.1	73.9 ± 15.3	0.215
VA (l)	62	5.7 ± 2.5	5.6 ± 1.3	0.759

LVED, left ventricle end diastolic volume; LVESV, left ventricle end systolic volume; LVEF, left ventricle ejection fraction; E, early peak velocity by pulsed wave Doppler; A, late (atrial) peak velocity by pulsed wave Doppler; E/A, ratio of the early (E) to late (A) ventricular filling velocities; DT, deceleration time; E/e', ratio of the transmitral early peak velocity by pulsed wave Doppler and the early diastolic mitral annulus velocity by tissue Doppler; TAPSE, tricuspid annular plane systolic excursion; LAVi, left atrial volume index; PAPs, systolic pulmonary artery pressure; VC, vital capacity; FVC, forced vital capacity; FEV_1_, forced expiratory volume in 1s; PEF, Peak expiratory flow; DLCO, diffusing capacity of the lungs for carbon monoxide (data corrected for hemoglobin); VA: alveolar volume.

**Figure 1:**
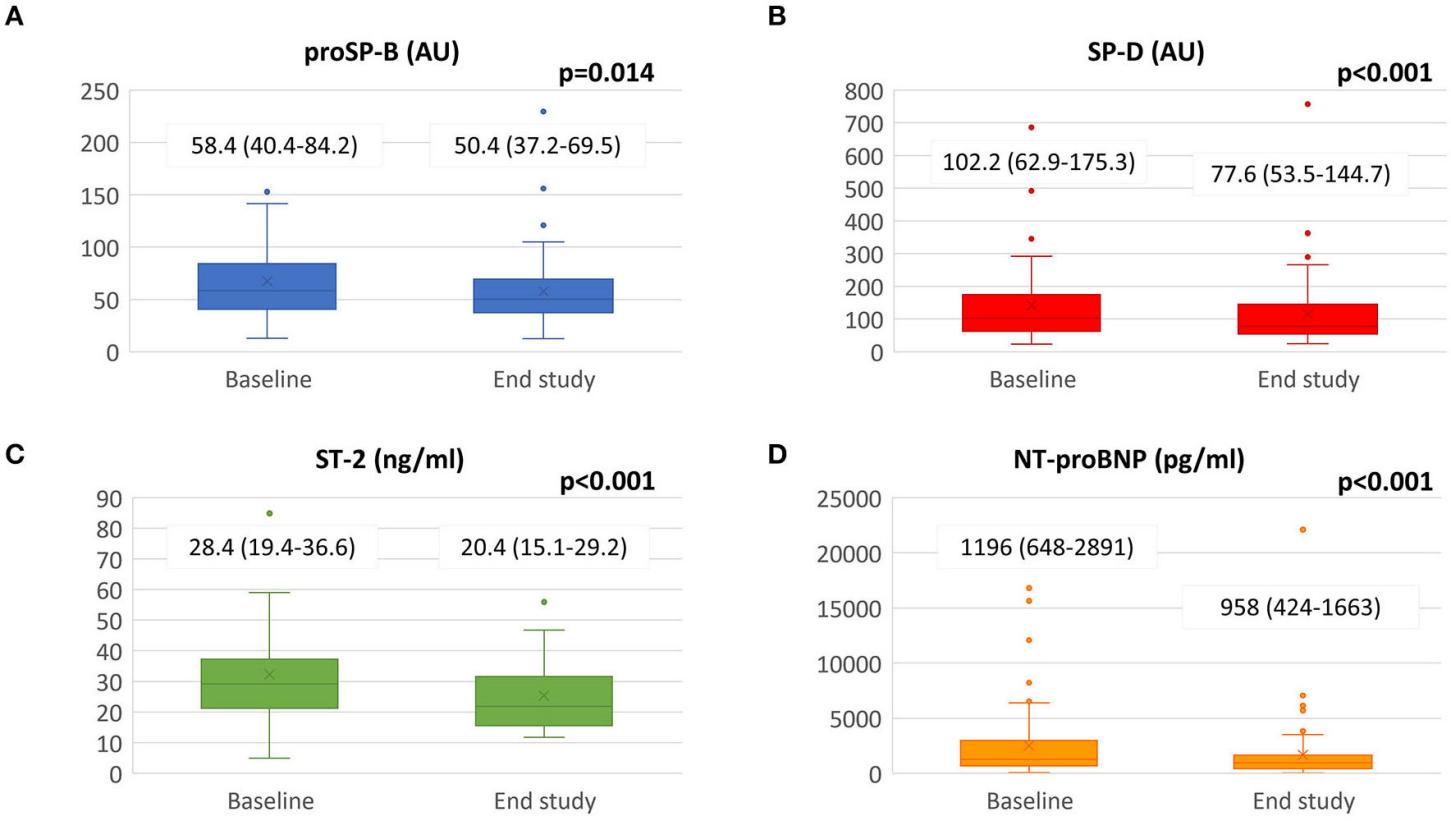
Biomarkers changes from baseline to the end of the study. Sacubitril/Valsartan significantly reduced panel **(A)** SP-B, panel **(B)** SP-D, panel **(C)** ST-2 and panel **(D)** NT-proBNP value after a median follow-up of 8.7 ± 1.4 months. Data presented as medians and interquartile ranges. proSP-B: immature form of surfactant protein isoform B; SP-D: surfactant protein isoform D; NT-proBNP: amino terminal pro-B-type natriuretic peptide; ST-2: interleukin ST-2.

As regards cardiac biomarkers NT-proBNP and interleukin ST-2, we observed a statistically significant reduction (Figure 1). Surfactant proteins also decreased: proSP-B from 58.43 AU [40.42–84.23] to 50.36 [37.16–69.54] (*p* = 0.014) and SP-D from 102.17 AU [62.85–175.34] to 77.64 [53.50–144.70] (*p* < 0.001).

Concerning pulmonary function test and lung diffusion measurement (Table 2), we observed a significant increase in VC and FEV_1_ but no changes in DLCO. However, when selecting only the patients with abnormal DLCO values at baseline (<80% of predicted) (*n* = 39), a significant improvement after treatment with Sacubitril/Valsartan (from 17.4 ± 4.1 to 18.9 ± 5.7 ml/min/mmHg; *p* = 0.006, corresponding to 65.3 ± 10.8 to 70.3 ± 15.9 % of predicted; *p* = 0.013) was observed.

**Figure 2:**
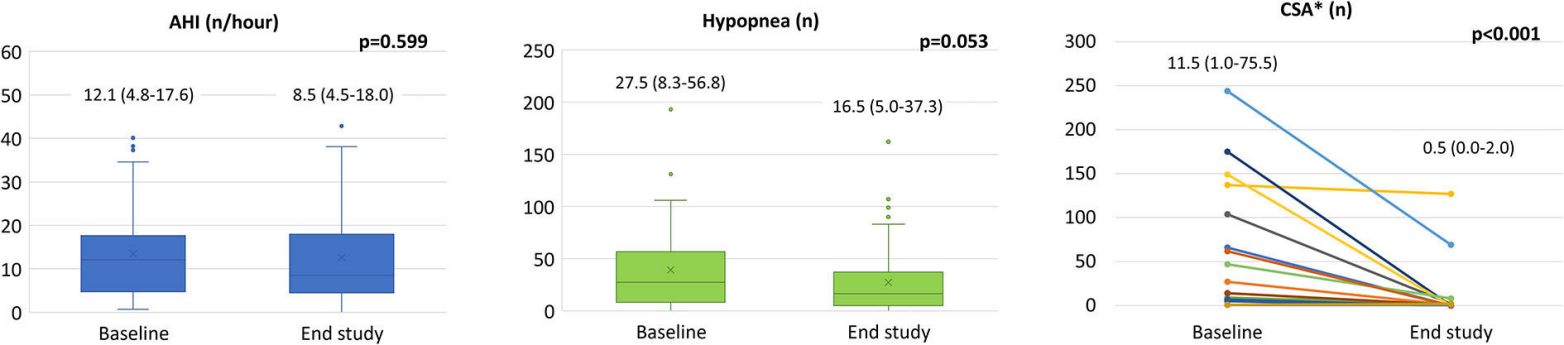
Sacubitril/Valsartan effects on sleep apneas. In the whole population we observed a significant reduction in CSA (see text) with no differences in AHI from baseline to the end of follow-up (left panel). In patients with CSA at baseline (*n* = 22) a significant reduction of CSA was obtained after Sacubitril/Valsartan therapy (right panel). Data presented as medians and interquartile ranges. AHI: apnea hypopnea index; CSA: central sleep apneas. *Analysis conducted only on patients with CSA>0 at baseline (*n* = 22).

Nocturnal cardiorespiratory monitoring was not performed in 15 patients due to technical reasons. In the remaining 56 patients, despite no significant changes in AHI, CSA and hypopnea events reduced from 0 [0–4] to 0 [0–1] (*p* = 0.027), and from 27.50 [8.25–56.75] to 16.50 [5.00–37.25] (*p* = 0.053), respectively (Figure 2). We did not observe significant changes in oxygen desaturation index after treatment with Sacubitril/Valsartan (89.5 ± 2.8 vs. 89.7 ± 2.0 %; *p* = 0.550). At least one CSA episode was present in 22 patients (39%) at baseline with an average of 11.5 [1.0–75.5] events. After Sacubitril/Valsartan treatment, CSA disappeared in 11 cases (50%) and reduced to 0.5 [0.0–2.0] (*p* < 0.001) (Figure 2). None of the patients without baseline CSA developed CSA at the follow-up. A further analysis was conducted in patients with AHI >15 (*n* = 20; 36%), showing similar results (AHI from 23.4 at baseline to 18.2 at the end of the study; *p* = 0.073; CSA from 7.5 at baseline to 0 at the end of the study; *p* = 0.003).

## Discussion

This prospective study explores the wide spectrum of Sacubitril/Valsartan effects on cardiac and pulmonary variables after 6 months of treatment at the maximum tolerated dose. Specifically, albeit Sacubitril/Valsartan has been extensively studied, we report, for the first time, its effects on DLCO, alveolar capillary released proteins and some HF biomarkers as ST-2, including them in a complete scenario of Sacubitril/Valsartan effects grouped as hemodynamic and pleiotropic ones. This comprehensive analysis represents the major novelty of the present study.

At the end of study, when more than 80% of patients reached the target dose of the drug (97/103 b.i.d.), we demonstrated an overall beneficial effect. In particular, in parallel with an improved alveolar capillary membrane diffusion capacity in patients with a baseline reduced DLCO, a reduction of surfactant proteins proSP-B and SP-D was observed. Conversely, we reported a significant reduction in biomarkers traditionally associated with HF (NT-proBNP and ST-2), in sleep apneas, as well as an improvement in left ventricular end-diastolic and end-systolic volumes, pulmonary pressures and left ventricular diastolic function parameters (Table 2 and Figure 2). The favorable effect of Sacubitril/Valsartan on cardiac remodeling, functional class, and natriuretic peptides is well-known; however, the mechanisms underlying these outcomes are less well understood, leaving room for two possible opposing theses: a predominantly hemodynamic effect or a predominantly non-hemodynamic effect, the so called systemic or pleiotropic (πλειóς = multiple, τρoπoς = change) effect.

### Evidences for a predominantly hemodynamic effect

A positive hemodynamic effect of Sacubitril/Valsartan is clearly shown by amelioration of cardiac ultrasound parameters such as improvement of left ventricle ejection fraction, reduction of left ventricle volumes, PAPs and E/e' values at the end of study (Table 2) (3). In addition, NT-proBNP, a BNP precursor secreted by the left ventricle in response to shear stress, is a biomarker strongly linked to the hemodynamic burden and its changes are related to the degree of patient's congestion, with higher NT-proBNP values observed in subjects with increased cardiac filling pressure. Moreover, it rapidly decreases in response to therapy primarily aimed to decongest HF patient (i.e., diuretics). Therefore, NT-proBNP significant reduction speaks in favor of the hemodynamic effect of Sacubitril/Valsartan (2).

Immature proSP-B, assayed in peripheral blood, is a novel biomarker in HFrEF and correlates with patients' prognosis (6). Increased circulating proSP-B values represent an indicator of alveolar cell stress, dysfunction, or even its death. Its immature form, contained within pneumocytes, is not found in the blood of healthy individuals, while it is released into the bloodstream in relation to hemodynamic stress on the alveolar capillary membrane such as episodes of HF (8), extra-deep diving (27), or invasive ventilation (4, 28, 29). Importantly, its values respond to hemodynamics targeted HF treatments, such as infusion of Levosimendan in acute HF (8), suggesting that it may be useful in the evaluation of treatment responsiveness. Thus, also the reduction in circulating proSP-B levels in response to Sacubitril/Valsartan is conceivable with a hemodynamic improvement.

Looking into lung function tests, FEV_1_ and VC are also indirect signs of congestion in HF (30) and their decrease a sign of extravascular lung fluid and cardiac size reduction. In fact, it has been demonstrated that HF patients develop pulmonary function abnormalities, ranging from minimal restriction to a severe restrictive pattern (31–34). From a pathophysiological point of view, in HF, the backward transmission of elevated left-sided filling pressure leads to pulmonary congestion, which may precede clinical signs of cardiac decompensation as shown in a trial involving prolonged invasive pulmonary pressures measurements (35). As left ventricular filling pressure increases, pulmonary congestion and interstitial oedema develop, causing reductions of lung volumes and compliance as assessable by spirometry. Therefore, the significant improvement in FEV_1_ and VC we detected following Sacubitril/Valsartan, likely showcases again a drug-induced hemodynamic improvement (36, 37).

Along with biomarkers and lung function tests, another parameter used to determine HF severity is the presence of sleep apneas, which are associated with unfavorable patients' prognosis. In our population, we have documented mild sleep disorders with a significant reduction in CSA, which disappeared in half of the patients who presented CSA at baseline (Figure 2, middle panel). This result was in accordance with a recent study by Passino et al. (38) that also demonstrated a CSA-driven reduction in apnea events in a cohort of 51 patients treated with Sacubitril/Valsartan. We did not detect an improvement in AHI as they did, probably because our population had less severe HF, as noted by a lower NYHA class (18% in NYHA class III vs. 41% in their population) and baseline nighttime AHI (12 vs. 19 events/hour). Taken together, these polysomnography findings are in line with the hemodynamic improvement led by treatment with Sacubitril/Valsartan. Moreover, they confirm the previous data by Apostolo et al. (20) that demonstrated that a hemodynamic amelioration in LVAD patients is correlated with a reduction in the number of apneas and specifically CSA. Importantly, a mechanical strategy based on nocturnal continuous positive airway pressure treatment to reduce sleep apneas failed to improve HF patients' prognosis (39, 40) and, at present, no other disease-modifying HFrEF therapies have demonstrated to reduce CSA burden. Therefore, considering the key role of sleeping disorder in determining HFrEF prognosis and the crucial role of congestion in their genesis, this could be considered an ancillary hemodynamic-driven mechanism of clinical improvement in patients treated with Sacubitril/Valsartan.

In brief, improvement of left ventricle volume and performance as well as lung mechanics, reduction of NT-proBNP and CSA are all suggestive of a hemodynamic driven Sacubitril/Valsartan effect.

### Evidences for a predominantly pleiotropic effect

At a first glace the Sacubitril/Valsartan pleiotropic effects are more hidden but nevertheless present. Indeed, interleukin ST-2 is induced and released by stretched myocytes in response to ventricular wall stress, hence its dysregulated concentrations in HF patients reflect a more remodeled heart (10). However, ST-2 is not affected by an acute hemodynamic stress such as exercise (41). ST-2 has emerged as an additional biomarker for HF, given its contribution to the genesis of fibrosis and inflammatory response (10). We observed a reduction of almost one third in ST-2 levels following Sacubitril/Valsartan treatment, supporting the hypothesis of the potential antifibrotic and anti-inflammatory effect of the drug. This is in line with previous findings from an analysis of the PARADIGM-HF data, where a reduction in ST-2 values, as well as in other biomarkers associated with profibrotic signaling, was shown (42).

In chronic HF the alveolar capillary membrane dysfunction is associated with long-lasting pathophysiological mechanisms such as interstitial fibrosis, local thrombosis and an increased in cellularity on top of lung fluid increase. As previously described in other therapeutic settings (8, 36, 37), treatment with ultrafiltration or inotropes in acute HF patients, despite an important and rapid lung fluid reduction, opposite to lung mechanics, does not affect DLCO which remains unchanged even after hemodynamic amelioration (36, 37, 43, 44). The same lack of improvement in lung membrane diffusing capacity is also observed following heart transplantation (45). On the other side, long term treatment with drugs which marginally affect pulmonary hemodynamic is associated with either DLCO improvement (ACE-i and mineralcorticoid receptor antagonists) or worsening (unselective β-blockers) (46–48). The suggested mechanisms are alveolar β-2 receptor blockage for β-blockers, bradikinine increase for ACE-I, and an antifibrotic action for MRA (46–48). On the other side, no effect on DLCO is present with ARBs treatment. Therefore, the significant DLCO improvement detected in our HF patients with reduced DLCO suggests a reduction of alveolar capillary membrane fibrosis by sacubitril and valsartan combined. This is all the more relevant because, according to the standard of care in HFrEF, in 73% of our population Sacubitril/Valsartan replaced ACE-I therapy, thus potentially removing a baseline beneficial effect of the latter on lung diffusion. Indeed, no other treatment potentially affecting membrane function (i.e., β-blockers or mineralcorticoid receptor antagonists) has been modified during the study.

Moreover, looking into surfactant binding proteins, differently from proSP-B, which is mainly produced by the alveolar cells with no site of synthesis outside the lungs, isoform D (SP-D) is a non-lung specific biomarker playing a role in the resolution of inflammation (4). Therefore, its significant reduction as detected in our study, supports the anti-inflammatory hypothesis attributed to Sacubitril/Valsartan.

Finally, these evidences of an antifibrotic effect of Sacubitril/Valsartan are also supported by the significant enhancement of left ventricle function which is considered in the first place as a purely hemodynamic effect. However, disease-related left ventricular remodeling is a complex process involving cardiac myocyte growth and death, vascular rarefaction, fibrosis, and inflammation (49). In a recent analysis by Iborra-Egea et al. (50), Sacubitril/Valsartan was found to attenuate cardiomyocyte cell death, hypertrophy, and impaired myocyte contractility, *via* different complex molecular mechanisms, thus triggering a series of cascades that participate in cardiac remodeling (50). In our trial we confirmed the positive effect of Sacubitril/Valsartan on left ventricle volumes and systolic function in a population almost equally divided between ischaemic and non-ischaemic patients. All together the remodeling effects of Sacubitril Valsartan we observed do not appear to be justified only by a hemodynamic improvement.

**Figure 3:**
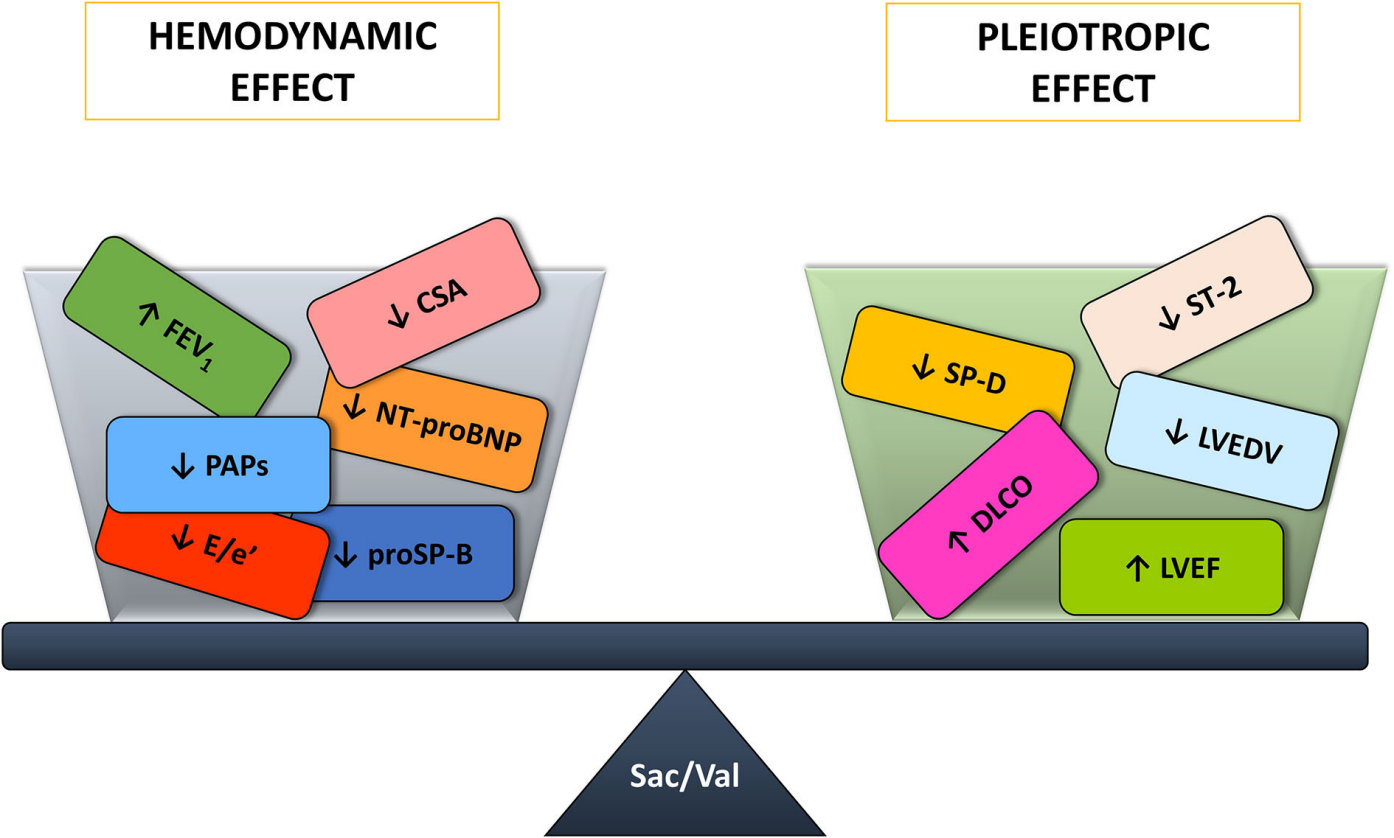
Sacubitril/Valsartan benefits on the cardiorespiratory system arise from the balance between the hemodynamic and pleiotropic effects. CSA, central sleep apneas; DLCO, diffusing capacity of the lungs for carbon monoxide; E/e', ratio of the transmitral early peak velocity by pulsed wave Doppler and the early diastolic mitral annulus velocity by tissue Doppler; FEV_1_, forced expiratory volume in 1s; LVEDV, left ventricle end diastolic volume; LVEF, left ventricle ejection fraction; NT-proBNP, amino terminal pro-B-type natriuretic peptide; PAPs, systolic pulmonary artery pressure; proSP-B, immature form of surfactant protein isoform B; Sac/Val, Sacubitril/Valsartan; SP-D, surfactant protein isoform D; ST-2, interleukin ST-2.

In the end, Sacubitril/Valsartan benefits are the result of a combination of hemodynamic and systemic effects with on one side an improvement in lung mechanics, congestion biomarkers, and sleep apneas and on the other the amelioration of lung diffusion, inflammation biomarkers and echocardiographic parameters (Figure 3). However, it is unknown whether the hemodynamic or the pleiotropic effects predominate and how they interact between each other. Indeed, a pleiotropic effect may trigger a hemodynamic one and vice versa.

### Study limitations

Our study, due to ethical reasons, lack of a randomized control group, however all our patients were in stable clinical condition, have been followed-up on a monthly basis and there were not significant changes in other HFrEF drugs.

This is a monocentric study and our results may not apply to other population, however another study recently published (38) showed similar results on sleep apneas. It should be taken into account that only a subgroup of our population completed the two nocturnal cardiorespiratory monitoring as per protocol.

The kinetics of the change in biomarkers (both lung surfactant proteins and ST-2 and NT-proBNP) during dose titration were not assessed in the present study. Therefore, it is not possible to understand how dose-dependent the effect is.

## Conclusion

In conclusion, taken together, our data demonstrate that, at least in the medium term (6 months), Sacubitril/Valsartan effect shares a double pathway: hemodynamic and systemic. The first is evidenced by the improvement in lung mechanics, reduction in NT-proBNP and immature proSP-B, and significant decrease in CSA. The latter is confirmed by an amelioration of lung diffusion, ST-2 and SP-D values as well as by echocardiographic parameters of positive reverse remodeling.

## Data availability statement

The datasets presented in this study can be found in online repositories. The names of the repository/repositories and accession number(s) can be found at: https://zenodo.org/, 6779121.

## Ethics statement

The studies involving human participants were reviewed and approved by Comitato Etico IRCCS Istituto Europeo di Oncologia e Centro Cardiologico Monzino. The patients/participants provided their written informed consent to participate in this study.

## Author contributions

Material preparation, data collection, and analysis were performed by MM, IM, ES, CBan, SG, FD, PG, VM, VV, VS, and BN. The first draft of the manuscript was written by MM, IM, ES, and PA. All authors contributed to the study conception and design, commented on previous versions of the manuscript, read, and approved the final manuscript.

## Funding

The present study was supported by the Italian Ministry of Health (Ricerca Corrente).

## Conflict of interest

The authors declare that the research was conducted in the absence of any commercial or financial relationships that could be construed as a potential conflict of interest.

## Publisher's note

All claims expressed in this article are solely those of the authors and do not necessarily represent those of their affiliated organizations, or those of the publisher, the editors and the reviewers. Any product that may be evaluated in this article, or claim that may be made by its manufacturer, is not guaranteed or endorsed by the publisher.
